# Supplementary material for: Evaluating methods for genome sequencing of Chlamydia trachomatis and other sexually transmitted bacteria directly from clinical swabs
Source: Microb Genom. 2025 Feb 13;11(2):001353. doi: 10.1099/mgen.0.001353 (PMC12282229; doi:10.1099/mgen.0.001353)
Supplement: Uncited Supplementary Material 1. [file mgen-11-01353-s001.pdf]

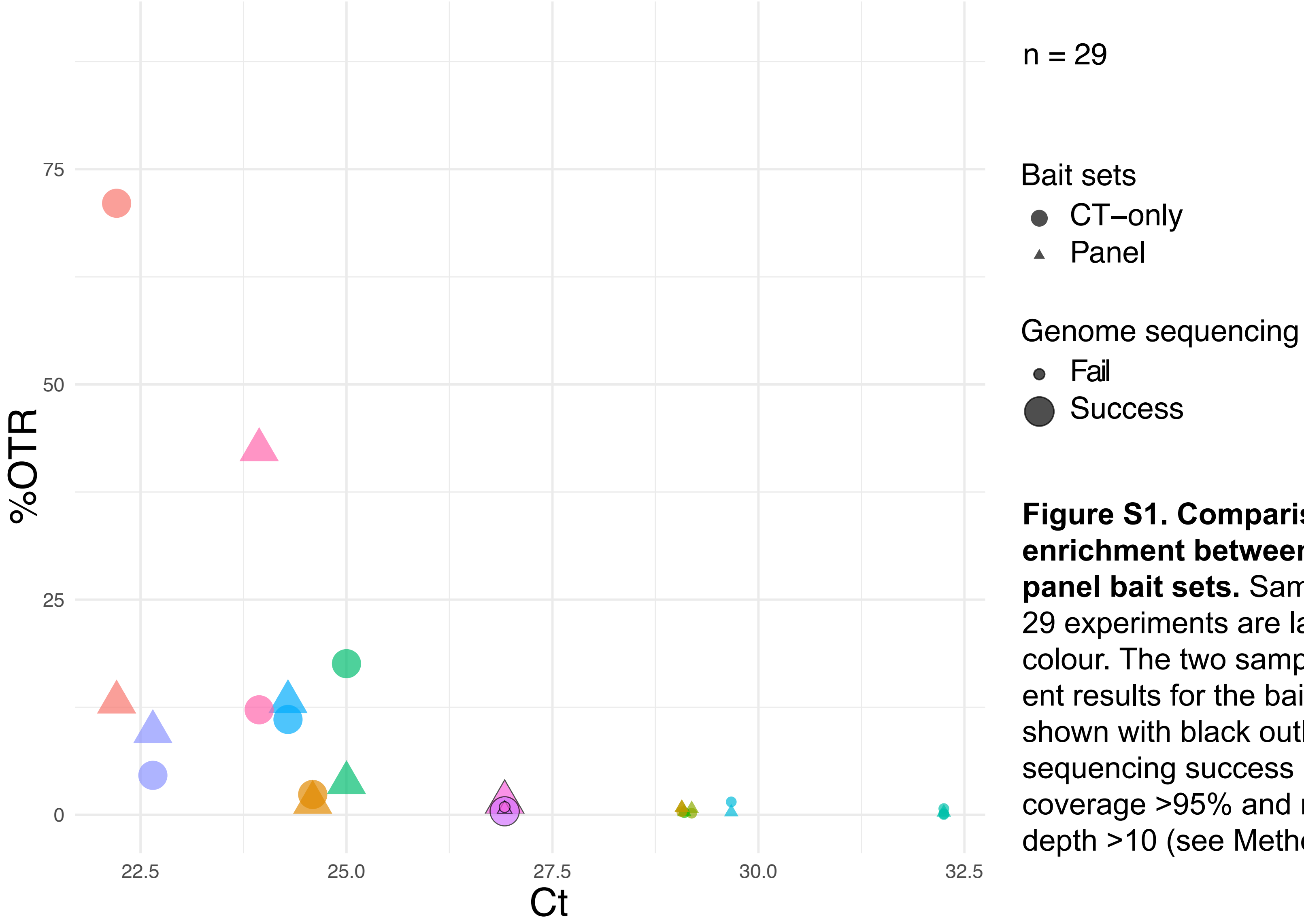

Figure S1. Comparison of CT data enrichment between CT-only and panel bait sets. Samples (13) over 29 experiments are labelled by colour. The two samples with different results for the baits sets are shown with black outlines. Genome sequencing success is defined as coverage >95% and mean read depth >10 (see Methods).
